# Psychotropic medication use and its metabolic consequences: advocating for a patient-centred approach

**DOI:** 10.1192/j.eurpsy.2025.2166

**Published:** 2025-08-26

**Authors:** L. Young, R. Rasheed

**Affiliations:** 1 Chapel street surgery, Rigg Milner Medical and Corringham health centre, and Collingwood Surgery, Essex, United Kingdom

## Abstract

**Introduction:**

There exponential rise in prescribing SSRIs and antipsychotic medication have highlighted the paucity of shared decision making, holistic therapies and weight management programmes. Guidelines are vague about checkpoints and deprescribing, seeming to ignore the metabolic consequences, failing to advocate for physical health monitoring.

**Objectives:**

We undertook a retrospective analysis of (n =492) patients initiated on psychotropic medication to understand the physical consequences, assessing the metabolic consequences of long-term prescribing of psychotropic medication in a rural primary care population.

**Methods:**

Electronic patient records were analysed from depression and serious mental health registers across three primary care sites. Data collected included the start date of treatment, BMI measurements before and after treatment.

**Results:**

Among the 220 patients studied, 150 (67.7%) experienced weight gain, while 70 (31.8%) lost weight. The average treatment duration was 11.5 years. Weight gain was more prevalent among males (71.6%) compared to females (66.7%). Patients who gained weight were, on average, prescribed cumulatively more psychotropic medications (3.21) than those who lost weight (2.89).

**Conclusions:**

Shared decision making and acknowledgement of the metabolic consequences of these medications at the point of initiation, and identification of pre-existent metabolic risks, monitor weight gain, and ensure adequate life-style changes to mitigate side effects. These methods should continue throughout the duration of treatment, and when possible, the option of deprescribing should always be available to the patient. It is time care bundles for patients with mental health illness are resourced for primary care.

**Disclosure of Interest:**

None Declared

